# Human Adenovirus B7d–Associated Urethritis after Suspected Sexual Transmission, Japan

**DOI:** 10.3201/eid2610.191538

**Published:** 2020-10

**Authors:** Nozomu Hanaoka, Shin Ito, Naomi Nojiri, Masami Konagaya, Mitsuru Yasuda, Takashi Deguchi, Tsuguto Fujimoto

**Affiliations:** National Institute of Infectious Diseases, Tokyo, Japan (N. Hanaoka, N. Nojiri, M. Konagaya, T. Fujimoto); i; Clinic, Sendai City, Japan (S. Ito);; Gifu University Hospital, Gifu City, Japan (M. Yasuda);; Kizawa Memorial Hospital, Gifu City (T. Deguchi)

**Keywords:** human adenovirus infections, urethritis, epidemiology, viruses, HAdV-B7d, Japan

## Abstract

Outbreaks of acute respiratory disease associated with human adenovirus (HAdV) B7d have been reported, including fatal cases in the United States. In 2018, we detected HAdV-B7d in a patient with urethritis, probably transmitted through sexual contact. Infectious HAdV-B7d was excreted in urine and gargle for >10 days after the disappearance of symptoms.

Human adenoviruses (HAdVs) are DNA viruses that can cause respiratory diseases, conjunctivitis, and gastroenteritis (*1*). Seven species (A–G) and *>*100 types have been recognized so far. Among them, HAdV-E4, HAdV-B7, and HAdV-B14 cause severe acute respiratory illness, including severe acute respiratory distress syndrome (*2*). HAdV-B7d, a genome type of HAdV-B7, was originally designated in 1986 using restriction analysis (*3*) and classified as genotype B7d on the basis of complete genome analysis in 2013 (*4*). HAdV-B7d was first reported in China in 1980 (*3*); by the 1990s, HAdV-B7d was the primary circulating genome type in China, but then it was not detected during 1990–2009. In 2011, HAdV-B7d was prevalent throughout Asia, and outbreaks of infant pneumonia related to HAdV-B7d were reported in China (*5*–*7*).

In Japan, routine national surveillance for HAdVs is conducted for epidemic keratoconjunctivitis, pharyngeal conjunctival fever, and infectious gastroenteritis, and reported in the *Infectious Diseases Weekly Report* (*8*). Outbreaks of HAdV-B7d, including 2 fatal cases, were observed in Japan during 1995–1996 (*9*), after which it was rarely detected until the occurrence of the case we describe in 2018 (*10*).

In 2014, HAdV-B7d was detected in Oregon and Illinois, USA (*11**,**12*). During 2016–2017, a total of 12 cases were reported, including 3 patients in a residential rehabilitation center, 7 college students, and 2 patients at a tertiary care hospital. Four of those 12 case-patients died; all 4 were in 3 adjacent New Jersey counties and had underlying conditions (*2*,*13*,*14*). In 2018, an HAdV-B7d outbreak at multiple facilities in several US regions resulted in the deaths of 11 infants at rehabilitation center in New Jersey and an 18-year-old freshman at the University of Maryland (*15*). In summary, HAdV-B7d transmission occurred in community and congregate settings throughout the United States, resulting in severe illness and death in some patients with underlying conditions. HAdV-B7d has been more commonly associated with severe respiratory disease and has a higher mortality rate than other HAdV types (*6*,*12*). Therefore, clinicians and public health facilitators should consider HAdV-B7d in patients with severe respiratory infections.

## The Study

Since 2013, we have focused on HAdV-associated urethritis and performed pathogen screening from the urine of all-male patients with acute urethritis at iClinic in Sendai City, Miyagi Prefecture, Japan; all patients gave informed consent (reference *16* in Appendix). Recently, several papers have reported that HAdVs are ranked the third- or fourth-highest causative agents of nonchlamydial, nongonococcal urethritis (reference *17* in Appendix). Furthermore, HAdVs most commonly associated with urethritis are those that cause epidemic keratoconjunctivitis, which are types D37, D56, and D64 (references *16,18* in Appendix).

**Figure 1:**
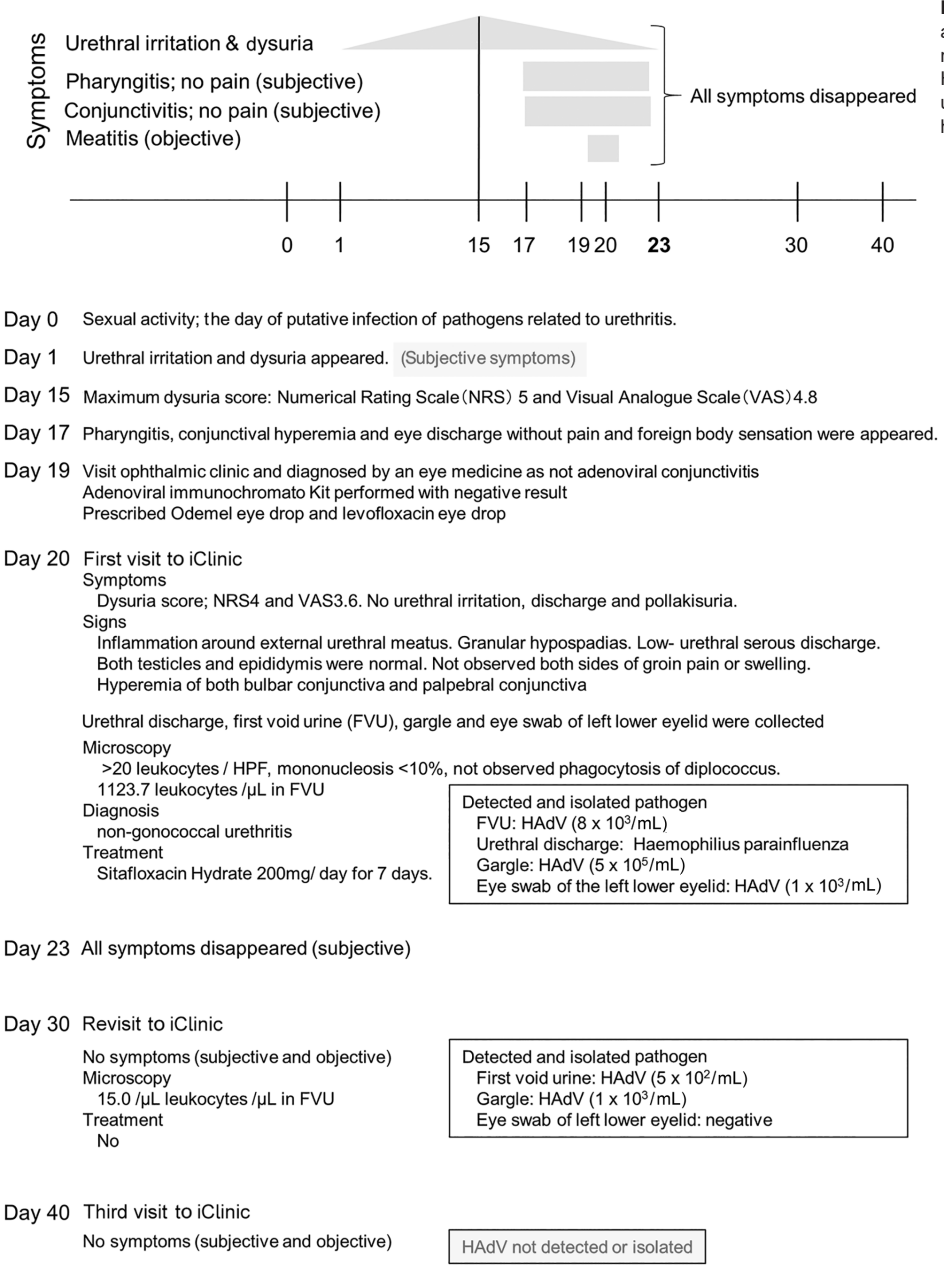
Clinical course and laboratory test results for patient with HAdV B7d–associated urethritis, Japan. HAdV, human adenovirus

In July 2018, a case of male urethral inflammation associated with HAdV-B7d was detected in a 22-year-old heterosexual man. He was unmarried and did not have a specific sexual partner. He had an unremarkable medical history, no history of sexually transmitted infections, and no record of traveling abroad. He claimed to have had 2 sexual encounters during his lifetime; the first was in 2016, but the second, in 2018, we considered to be the putative infection day (day 0) (Figure 1). He described it as a casual sexual encounter with a previously unknown woman and reported the encounter to include protected vaginal intercourse, cunnilingus, and unprotected oral intercourse. The patient denied insertive or receptive anal intercourse as well as mouth-to-mouth kissing. Urethral irritation and dysuria appeared on day 1 and continued to develop gradually (Figure 1); these symptoms might be caused by mechanical irritation. On day 15, the patient experienced the most pain from his symptoms, reporting a numerical rating scale score of 5/10 and a visual analog scale score of 4.8/10. These scales are subjective scores of pain (reference *19* in Appendix). Pharyngitis and conjunctivitis appeared on day 17. On day 19, he visited an ophthalmic clinic for confirmation of conjunctivitis; however, it was not considered to be adenoviral conjunctivitis because an adenoviral immunochromatographic kit produced a negative result. Fluorometholone and levofloxacin eye drops were prescribed. Because his urethritis symptoms were severe, he visited a sexually transmitted diseases clinic (Sendai city, Miyagi prefecture, Japan) on day 20. The patient reported no fever, chills, or malaise; on the basis of his symptoms and results of a physical examination (Figure 1), we diagnosed nongonococcal urethritis. Because we could not exclude the possibility of bacterial infection, we prescribed sitafloxacin hydrate (200 mg/d for 7 days). Pathogen screening at the first visit to the clinic detected *Haemophilus parainfluenzae* bacteria from urethral discharge, but the clinical significance was unclear.

**Figure 2:**
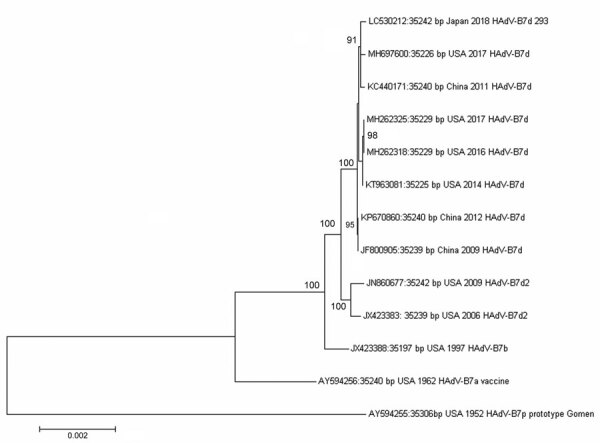
Phylogenetic analysis of human adenovirus genome type 7 whole-genome sequencing in study of human adenovirus B7d–associated urethritis, Japan. We isolated 293 strain and compared it with other human adenovirus type 7 reference strains. We aligned genomic sequences using ClustalW (http://www.clustal.org) and constructed the neighbor-joining phylogenetic tree using MEGA version 7.0 software (https://megasoftware.net). Numbers at selected nodes indicate level of support using 1,000 bootstrap replicates. Scale bar indicates the estimated number of nucleotide substitutions per site. Sequence names are derived from the GenBank accession number, geographic location, and year of sample collection and virus type.

We isolated HAdV in A549 cells from first-void urine, throat gargle, and eye discharge fluid by a previously described method (reference *16* in Appendix). No other pathogens were identified. Sequences of the HAdV hexon, fiber, and penton open reading frames obtained by Sanger sequencing from all 3 specimen sources were identical. The full genome sequence was obtained from the urine isolate (designated strain 293) (Appendix Tables 1, 2) and deposited in GenBank (accession no. LC530212). We also performed a BLAST analysis (https://blast.ncbi.nlm.nih.gov/Blast.cgi) as previously described (*2*) with reference sequences of HAdV type 7 (Appendix Figure 1; Figure 2). On the basis of the phylogenetic tree analysis of whole-genome sequences, we classified the isolated HAdV-B7 strain into the same cluster as HAdV-B7d. In addition, we performed an in silico analysis of the genome using Restriction Analyzer software (http://www.molbiotools.com/restrictionanalyzer.html) by comparing the patterns of the 293 isolate with a reference HAdV-7d sequence and the following enzymes: *Bam*HI, BclI, BstEII, *Hpa*I, and *Sma*I (Appendix Figure 2). After these analyses, we identified the 293 isolate as HAdV-7d. On the basis of these results, we concluded that the patient acquired the HAdV-B7d infection, which caused urethritis, conjunctivitis, and pharyngitis, during sexual intercourse. All symptoms disappeared by day 23. When the patient revisited the clinic on day 30, he had no urethral symptoms. We detected HAdV-B7d strains isolated from first-void urine and gargle but not from eye discharge; no other pathogens tested in this study were detected. On day 40, the patient’s third visit to the clinic, no pathogens were detected. Approximately 2 months later, no symptoms were observed, and we confirmed a good prognosis.

## Conclusions

HAdVs infect mucous membranes and can infect the urethra. Documented cases of HAdV urethritis are most often associated with certain species D HAdVs that cause epidemic keratoconjuctivitis (references *16–18* in Appendix). Our finding of HAdV-B7d, a virus more commonly associated with acute respiratory infections, in this patient was unexpected. Although we identified *H. parainfluenza* from urethral discharge at the first clinic visit, we suspect that it may have contributed to but not caused the patient’s primary symptoms. The isolated HAdV-B7d strains in this study were excreted in urine and gargle for >1 week after all symptoms had disappeared (Figure 1), which suggests that HAdV-B7d infection may cause urethritis and involve viral shedding into urine.

Appendix Additional information about human adenovirus B7d–associated urethritis, Japan.
