# Supplementary material for: Human Adenovirus B7d–Associated Urethritis after Suspected Sexual Transmission, Japan
Source: Emerg Infect Dis. 2020 Oct;26(10):2444–7. doi: 10.3201/eid2610.191538 (PMC7510690; doi:10.3201/eid2610.191538)
Supplement: Appendix — Additional information about human adenovirus B7d–associated urethritis, Japan. [file 19-1538-Techapp-s1.pdf]

Article DOI: https://doi.org/10.3201/eid2610.191538

Human Adenovirus B7d–Associated Urethritis after Suspected Sexual Transmission, Japan

Appendix

Additional References

16. Hanaoka N, Ito S, Konagaya M, Nojiri N, Yasuda M, Fujimoto T, et al. Infectious human adenoviruses are shed in urine even after disappearance of urethral symptoms. PLoS One. 2019;14:e0212434. PubMed https://doi.org/10.1371/journal.pone.0212434

17. Ito S, Hanaoka N, Shimuta K, Seike K, Tsuchiya T, Yasuda M, et al. Male non-gonococcal urethritis: from microbiological etiologies to demographic and clinical features. Int J Urol. 2016;23:325–31. PubMed https://doi.org/10.1111/iju.13044

18. Hiroi S, Kawahata T, Furubayashi K. First isolation of human adenovirus type 85 by molecular analysis of adenoviruses in cases of urethritis. J Med Microbiol. 2020;69:265–9. PubMed https://doi.org/10.1099/jmm.0.001149

19. Hawker GA, Mian S, Kendzerska T, French M. Measures of adult pain: Visual Analog Scale for Pain (VAS Pain), Numeric Rating Scale for Pain (NRS Pain), McGill Pain Questionnaire (MPQ), Short-Form McGill Pain Questionnaire (SF-MPQ), Chronic Pain Grade Scale (CPGS), Short Form-36 Bodily Pain Scale (SF-36 BPS), and Measure of Intermittent and Constant Osteoarthritis

Pain (ICOAP). Arthritis Care Res (Hoboken). 2011;63(Suppl 11):S240–52. PubMed https://doi.org/10.1002/acr.20543

Appendix Table 1. Primer name and sequence used in study of human adenovirus B7d–associated urethritis, Japan.

Name	Sequence
Ad7_5'-1	TCTATTTAATATACCTTATAGATG
Ad7_5'-2	ACTCTTGAGTGCCAGCGAGAAGAG
Ad7_5'-3	AAGCTGCCAGCGATGTTTTTAAGTTGG
Ad7_5'-4	TAAAATTATGTCAGCTGCTGAGTG
Ad7_5'-5	ACAATCTTAGATTACTGGCCAGTGC
Ad7_5'-6	TAATAGATACACAAGATAAAGCAG
Ad7_5'-7	TCTTGCTACTGTGCATATCGTTTCAC
Ad7_5'-8	AGTATTTAGCCCTTATCTGACGGGC
Ad7_5'-9	TTGAGAACTCGGTGGATCTTTTCCAG
Ad7_5'-10	TCTGAAACATCATAGTTATGCTCC
Ad7_5'-11	AAAGAGTCTGTTGCAAGAGCTCGAGCC
Ad7_5'-12	AGGTCAGATCCGGCTCATCGGGGTC
Ad7_5'-13	TAGGAACGAGGAGGATTTGATATTGAC
Ad7_5'-14	TCTCGAACTGCCAGCGCGCGCTCATAGG
Ad7_5'-15	AGTACTCTTCGAGGGGAAACCCGTC
Ad7_5'-16	AGTGTTCGAGGGCCCATTCGTGCACG
Ad7_5'-17	TGACTTCCTTTGACGAGAAATTTCAG
Ad7_5'-18	TGGTACTGCGCCCTGAGAAGACTCG
Ad7_5'-19	AGAAATACATGATCCATCGTCTCAGC
Ad7_5'-20	ATCGTCTCAAGATCCACGGGATCTG
Ad7_5'-21	ATTGTGTAGGAGCAGTTGGCCATGAC
Ad7_5'-22	AGCCGCAGCTAACGTGGTACTGGC
Ad7_5'-23	ACTGGCGCGCCTGGGCGCACCATCG
Ad7_5'-24	TAACTACTGCGGCTGCAGCCGTCAGC
Ad7_5'-25	ATCAGCCCAGCTAGGGCACATGTGG
Ad7_5'-26	ACTCTGAGCGACGATCTGGGGGTG
Ad7_5'-27	AACCCCACCCACGAGAAGGTCCTGG

Name	Sequence
Ad7_5'-28	TGCTGCTGCTGCTGGTATCC
Ad7_5'-29	AGTACGACATGCCCGACCCCAATG
Ad7_5'-30	TTGGGTGGAAGAGGAGGGGGCAACC
Ad7_5'-31	ATGGGGCGGTCATCTGAAGACCATC
Ad7_5'-32	ATGTATGAAGATCTTGAAGGGGG
Ad7_5'-33	AATGAGCAAGCCGTGTACTCTCAGC
Ad7_5'-34	TGCTCGTAATTATACTCCTACTGCGC
Ad7_5'-35	AAAAGCCCCGCAAAATCAAGCGGGTC
Ad7_5'-36	AGCCTGAGGTCAAAGTAAGACCTATC
Ad7_5'-37	ATATGGCCCTCACTTGCCGCCTTCG
Ad7_5'-38	AAATAAGCTTAAGGAACAAAACTTCC
Ad7_5'-39	TCTGAACAGCATCGTGGGTCTGGGC
Ad7_5'-40	TAGCTTCAAGCCATATTCCGGCAC
Ad7_5'-41	AATTTGGAAACTCCAGACAGCCATG
Ad7_5'-42	TTAATATCCAAGCTAATCTTTGGAG
Ad7_5'-43	ACTCTTGAAGCCATGCTGCGCAACG
Ad7_5'-44	AAACTTCCAGCCTATGAGCAGGCAGG
Ad7_5'-45	AGCTGCAGCCATGTCATGCGGGTC
Ad7_5'-46	AATCTCCCCAAGTGCAGCCCACCC
Ad7_5'-47	TGTCTCACGCTTGCCAGCACGGTCGG
Ad7_5'-48	TGCATCCATGAGCCCACAGAGCGC
Ad7_5'-49	TGGTCTTTCTGGGCTTCTTCTTGGG
Ad7_5'-50	AAGAGATTGAGGCAGATGTCGAGCAGG
Ad7_5'-51	TTACCTGATATAGCTTCCTTGGAAGAG
Ad7_5'-52	ACCTTTAGACATGGCTTCGTGCGG
Ad7_5'-53	ATGGAGCCACTGCTACCTGTTCCGC
Ad7_5'-54	AGGATGTCCCATCGCCGAGGAAGC
Ad7_5'-55	ATTACTACCGTCACCTCCACAGCC
Ad7_5'-56	TTGGCAGCAGGCGCCTCCCAGGAC
Ad7_5'-57	TCTCCAGTTCGTGGAGGAGTTTACTCC
Ad7_5'-58	ACCAACCAGATCTTCCAGAAGACCC
Ad7_5'-59	ACTGCAGGTCCGTTGAAATTACAC
Ad7_5'-60	ATTTGGACCAAACTTGGAAGTGTTG
Ad7_5'-61	TTGGCTCAACATGTACACTACAAGG

Name	Sequence
Ad7_5'-62	TATAGAATTATGATATTGTTTCAATC
Ad7_5'-63	ACTTCTTAGGCTTATTTAAAACCATGC
Ad7_5'-64	AACTACGCATCCGCCAGCAGCAGG
Ad7_5'-65	TGTACCCACAATCTTCATGTCTTTC
Ad7_5'-66	TAATTCTAACACTAGTTAAAACTGG
Ad7_5'-67	ACGACTGACAAATAAAGTTTAACTTG
Ad7_5'-68	ACTTAGATTACTACAGTAGGTACAGC
Ad7_5'-69	ACGAACACAACTTACACTATGCATAG
Ad7_5'-70	TAGATCGCGCAGATGGCATCTATCG
Ad7_5'-71	TACAAGCGCAGACCTCCCCAATTGG
Ad7_5'-72	ATGCTTAATCTTAAGTATAGCAAAGCC
Ad7_5_P7900	TTGTACAGACGGCCGCAGTACTCGC
Ad7_3_P9070	TTTTCAACTTTGCCGTGGACTTCTAC
Ad7_3'-1	TCTATATAATATACCTTATAGATGG
Ad7_3'-2	ATACTTAAGATTAAGCATAATTATACC
Ad7_3'-3	TGTAGCGTCCCCTGCTATTGTTCCC
Ad7_3'-4	ATTTGAAGTACTGCGAGATCGTTTGG
Ad7_3'-5	TTCCTTGTTCTGCCAGCTTTACTGTTC
Ad7_3'-6	TCAGAATGCGTTGCTGCGCGCACC
Ad7_3'-7	TCTAATGTAGTAAAAGGTAAATGGAG
Ad7_3'-8	ATTCACTGGAGGCCATTATTTGACAG
Ad7_3'-9	TTTGACATCCCCTTTAAAGTATGGAG
Ad7_3'-10	TTTGCATTGGTGAATTTGGATGAC
Ad7_3'-11	ATTTTGATTGCGGTATTCGGGATGG
Ad7_3'-12	ATGTCAAAATTTAGTAATGCATCAC
Ad7_3'-13	ATAAGTTACAGCTGCAAGGCTAGTAATG
Ad7_3'-14	AACCTCTGAAGTAATTGGGGGCCC
Ad7_3'-15	ACTTAATAAGAACTCCACAGAGACG
Ad7_3'-16	TTGTAAAGTCCTGATTGAAGAAGCGG
Ad7_3'-17	TTCCGATGCCGCCCGAGCGGGGC
Ad7_3'-18	ATTTGGGGCTGATAGCTCCACATG
Ad7_3'-19	AAGTTCACACCGTGGTGGAAGAGCAG
Ad7_3'-20	AACCTTGTGTTGAGCTCCTCACCGG
Ad7_3'-21	AGAGGCACAAAGTCGGAGGGCAGCG

Name	Sequence
Ad7_3'-22	TAGTGCAGGTTCTCCTCTAGCTTGC
Ad7_3'-23	AGCTCCCGGACCCAAGTTGAGAAGGG
Ad7_3'-24	TTTCACATTATCCTGCGCCTGCATC
Ad7_3'-25	AGCATGTCCCTCTGCAAGACATCGGC
Ad7_3'-26	ATTATGAAGGCAAGGTGAAATGCC
Ad7_3'-27	TTCCAGTGTTGCAACCCAGTGTACCG
Ad7_3'-28	TTTATTGAACACGGTTTTACATGAC
Ad7_3'-29	AAGCAAGAGGCTTCTTATGTGGTGGC
Ad7_3'-30	AAAAAGGAGTACATGCGATCCTTG
Ad7_3'-31	TGAAGCGGTGTTGTGAGCCATGGG
Ad7_3'-32	TTTCCATAGCCAGATTGTTGCCTATGG
Ad7_3'-33	TTCCGTGTAAAGCACAATTTCAGGCG
Ad7_3'-34	TAATGTCAAAGAATGTGCTGGCCATG
Ad7_3'-35	AGACCCACGATGCTGTTCAGAGTAC
Ad7_3'-36	AAGCTTTGTTCCCATAGGTTTTTACGG
Ad7_3'-37	AGGCAGCGTCAACAGTCATTAAGTGG
Ad7_3'-38	ATTTTCTCCAGTACGTCTTCTAGCC
Ad7_3'-39	TTTCATCCTTCACCGGTGGACCGTAG
Ad7_3'-40	TCGGCGACCACCTGGTCGATCACATC
Ad7_3'-41	TAGAAACTCTTTGAGAAGACGGGC
Ad7_3'-42	TTTGAAACCCTCCTGGAATGGATGTC
Ad7_3'-43	TTCGTCAAAGTTGATGGTCTGGGTG
Ad7_3'-44	TCCAACACGCTGCTATCATCGGCAG
Ad7_3'-45	AAACCAGGTGGGGGCAGCCAGTGTG
Ad7_3'-46	TTGGGAGTCAGCAAGCTAGACACGGTC
Ad7_3'-47	AAAGGCGGTTGGCCTGGGGTTGCTG
Ad7_3'-48	AGAGTCATGCGCATGTAAAACCCATC
Ad7_3'-49	TCCTCGTCTTGCAGCACCCGTCTTCG
Ad7_3'-50	AGTAGTTACAGGAGCAGGAAGAGCC
Ad7_3'-51	TCTTCCAATTCCAGATCATAGGCGG
Ad7_3'-52	TGATCCGAGATTCGAACCGGGGTAC
Ad7_3'-53	TTTGTGGACTTTGACGACTTCCAAG
Ad7_3'-54	TTGCGCGGAGTACCTACGGGGCAATTG
Ad7_3'-55	TCGTCACCGAGCACATCGCCACCAC

Name	Sequence
Ad7_3'-56	AACCTACCCGCGCGCGGCGCCGAC
Ad7_3'-57	ATTCATGAGGTGCATCCCGTGAATCG
Ad7_3'-58	TGCTCTCACTGACCCTACAGATCTCAC
Ad7_3'-59	AAGGATCGCGAAGAATACCTTCTC
Ad7_3'-60	AGATGACCTTGGATGATCCCACCACC
Ad7_3'-61	AACTGACAACCTGAGTGCAGAGGTC
Ad7_3'-62	AAACCGTCTGCGCCTCCTGCGGTGCG
Ad7_3'-63	AAATGCCCATGGCTGACGGGCTGAAG
Ad7_3'-64	TATCATTATGGATGAGTGCATGGAG
Ad7_3'-65	TGATCGAGACCGATGGTCCAGGGC
Ad7_3'-66	AAGCGCTTCCACTCATGGCAGCTGC
Ad7_3'-67	ATATTGCAATGTCCACCAGCGCAGG
Ad7_3'-68	ATTCCCTGATATGTAGCATGC
Ad7_3'-69	TCATCCTGCGAGCCTTCCATGTTC
Ad7_3'-70	TACATGACGTCACATTAAATAAACAC
Ad7_3'-71	ATTCACTGCGGTATGGATGGACTGCTC
Ad7_3'-72	AACTCGTCAGGTTTAAATACCCTAGCG
Ad7_5_39_2	ATGCTTCGGAGTACCTGAGTCCGG
Ad7_5_40_2	ATATCAGCCAGAGCCTCAAGTTGG
Ad7_3_34_2	TTTCATTTGTTCCATCAATATCAG
Ad7_3_33_2	TTCAGTGTTTCTGTCCTGCAAGTC
Ad7_up_3_1*	TTTTAGCCGTTCACCCCACAGCC
Ad7_up_3_2*	AATTTTTACTTGCATCCGCC
Ad7_down_5_1*	TTTTTTAAATTACCTCATTTGC
Ad7_down_5_2*	AGGGAAAAGTACAGTTTCACTTCC

*5' and 3' end of 293 genome was determined by direct sequencing using Ad7_up3_1, 3_2, Ad7_down_5_1 and 5_2 with its genomic DNA.

Appendix Table 2. Primer set for PCR amplification and sequencing in study of human adenovirus B7d–associated urethritis, Japan*

Fragment	1-3500	3000-6500	5500-8000	6000-9500	7500-10000	9000-12500	12000-15500	15000-18500	18000-21500	21000-24500	24000-27500	27000-30500	30000-33500	33000-35000
PCR forward primer	Ad7_5'-1	Ad7_5'-7	5-12	Ad7_5'-13	5-16	Ad7_5'-19	Ad7_5'-26	Ad7_5'-32	Ad7_5'-38	Ad7_5'-44	Ad7_5'-50	Ad7_5'-56	Ad7_5'-62	Ad7_5'-68
PCR reverse primer	Ad7_3'-66	Ad7_3'-60	3-57	Ad7_3'-54	3-53	Ad7_3'-47	Ad7_3'-41	Ad7_3'-35	Ad7_3'-29	Ad7_3'-23	Ad7_3'-17	Ad7_3'-11	Ad7_3'-5	Ad7_3'-1
Sequencing primer	Ad7_5'-1	Ad7_5'-7	Ad7_5'-12	Ad7_5'-13	Ad7_5'-16	Ad7_5'-19	Ad7_5'-26	Ad7_5'-32	Ad7_5'-38	Ad7_5'-44	Ad7_5'-50	Ad7_5'-56	Ad7_5'-62	Ad7_5'-68
	Ad7_5'-2	Ad7_5'-8	Ad7_5'-13	Ad7_5'-14	Ad7_5'-17	Ad7_5'-20	Ad7_5'-27	Ad7_5'-33	Ad7_5'-39	Ad7_5'-45	Ad7_5'-51	Ad7_5'-57	Ad7_5'-63	Ad7_5'-69
	Ad7_5'-3	Ad7_5'-9	Ad7_5'-14	Ad7_5'-15	Ad7_5'-18	Ad7_5'-21	Ad7_5'-28	Ad7_5'-34	Ad7_5_39_2	Ad7_5'-46	Ad7_5'-52	Ad7_5'-58	Ad7_5'-64	Ad7_5'-70
	Ad7_5'-4	Ad7_5'-10	Ad7_5'-15	Ad7_5'-16	Ad7_5'-19	Ad7_5'-22	Ad7_5'-29	Ad7_5'-35	Ad7_5'-40	Ad7_5'-47	Ad7_5'-53	Ad7_5'-59	Ad7_5'-65	Ad7_5'-71
	Ad7_5'-5	Ad7_5'-11	Ad7_5'-16	Ad7_5'-17	Ad7_5'-20	Ad7_5'-23	Ad7_5'-30	Ad7_5'-36	Ad7_5_40_2	Ad7_5'-48	Ad7_5'-54	Ad7_5'-60	Ad7_5'-66	Ad7_5'-72
	Ad7_5'-6	Ad7_5'-12	Ad7_3'-57	Ad7_5'-18	Ad7_5_P7900	Ad7_5'-24	Ad7_5'-31	Ad7_5'-37	Ad7_5'-41	Ad7_5'-49	Ad7_5'-55	Ad7_5'-61	Ad7_5'-67	Ad7_3'-1
	Ad7_5'-7	Ad7_5'-13	Ad7_3'-58	Ad7_5'-19	Ad7_3'-53	Ad7_5'-25	Ad7_5'-32	Ad7_5'-38	Ad7_5'-42	Ad7_5'-50	Ad7_5'-56	Ad7_5'-62	Ad7_5'-68	Ad7_3'-2
	Ad7_3'-66	Ad7_3'-60	Ad7_3'-59	Ad7_3'-54	Ad7_3'-54	Ad7_5'-26	Ad7_3'-41	Ad7_3'-35	Ad7_5'-43	Ad7_3'-23	Ad7_3'-17	Ad7_3'-11	Ad7_3'-5	Ad7_3'-3
	Ad7_3'-67	Ad7_3'-61	Ad7_3'-60	Ad7_3'-55	Ad7_3'-55	Ad7_3'-47	Ad7_3'-42	Ad7_3'-36	Ad7_5'-44	Ad7_3'-24	Ad7_3'-18	Ad7_3'-12	Ad7_3'-6	Ad7_3'-4
	Ad7_3'-68	Ad7_3'-62	Ad7_3'-61	Ad7_3'-56	Ad7_3'-56	Ad7_3'-48	Ad7_3'-43	Ad7_3'-37	Ad7_3'-29	Ad7_3'-25	Ad7_3'-19	Ad7_3'-13	Ad7_3'-7	Ad7_3'-5

Fragment	1-3500	3000-6500	5500-8000	6000-9500	7500-10000	9000-12500	12000-15500	15000-18500	18000-21500	21000-24500	24000-27500	27000-30500	30000-33500	33000-35000
	Ad7_3'-69	Ad7_3'-63		Ad7_3'-57	Ad7_3'-57	Ad7_3'-49	Ad7_3'-44	Ad7_3'-38	Ad7_3'-30	Ad7_3'-26	Ad7_3'-20	Ad7_3'-14	Ad7_3'-8	
	Ad7_3'-70	Ad7_3'-64		Ad7_3'-58	Ad7_3_P9070	Ad7_3'-50	Ad7_3'-45	Ad7_3'-39	Ad7_3'-31	Ad7_3'-27	Ad7_3'-21	Ad7_3'-15	Ad7_3'-9	
	Ad7_3'-71	Ad7_3'-65		Ad7_3'-59		Ad7_3'-51	Ad7_3'-46	Ad7_3'-40	Ad7_3'-32	Ad7_3'-28	Ad7_3'-22	Ad7_3'-16	Ad7_3'-10	
	Ad7_3'-72	Ad7_3'-66		Ad7_3'-60		Ad7_3'-52	Ad7_3'-47	Ad7_3'-41	Ad7_3'-33	Ad7_3'-29	Ad7_3'-23	Ad7_3'-17	Ad7_3'-11	
						Ad7_3'-53			Ad7_3_33_2					
						Ad7_3'-54			Ad7_3'-34					
									Ad7_3_34_2					
									Ad7_3'-35					

*PCR was performed with GXL enzyme [TAKARA BIO, Kyoto, Japan] as previously described (Biggs et al. [*14*]). DNA fragments was purified and performed sequencing with indicated primers by FASMAQ Company, http://fasmac.co.jp.

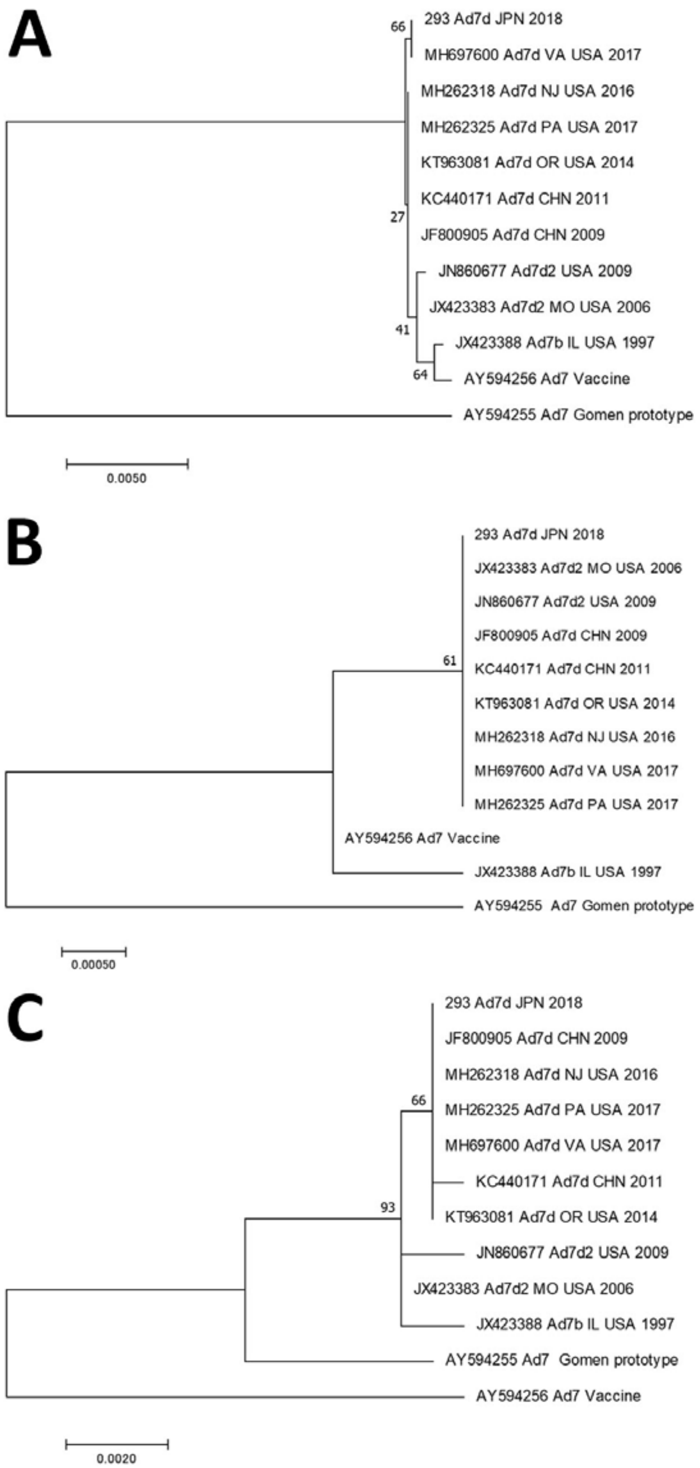

Appendix Figure 1. Molecular phylogenetic analysis of human adenovirus 293 strain isolated in this study, compared with other human adenovirus type 7 reference strains. We aligned hexon (A), fiber (B) and penton (C) open reading frames using ClustalW (http://www.clustal.org) in

MEGA version 7 (https://www.megasoftware.net). We inferred the evolutionary history by maximum-likelihood method based on the Kimura 2-parameter model (A,C) or Tamura 3-parameter model (B). The tree with the highest log likelihood (A: –4462.55, B:-1392.74 and C: –2533.45) is shown. The percentage of trees in which the associated taxa clustered together is shown next to the branches. We obtained initial trees for the heuristic search automatically by applying neighbor-join and BioNJ (http://bionj.org) algorithms to a matrix of pairwise distances estimated using the maximum composite likelihood approach, and then selecting the topology with superior log likelihood value. The tree is drawn to scale, with branch lengths measured in number of substitutions per site. The analysis involved 12 nt sequences. All positions containing gaps and missing data were eliminated. The final dataset included a total of 2,805 positions (A), 978 positions (B), and 1,635 positions (C). We conducted evolutionary analyses in MEGA7. Sequence names are derived from the GenBank accession number, geographic location, year of sample collection, and virus type.

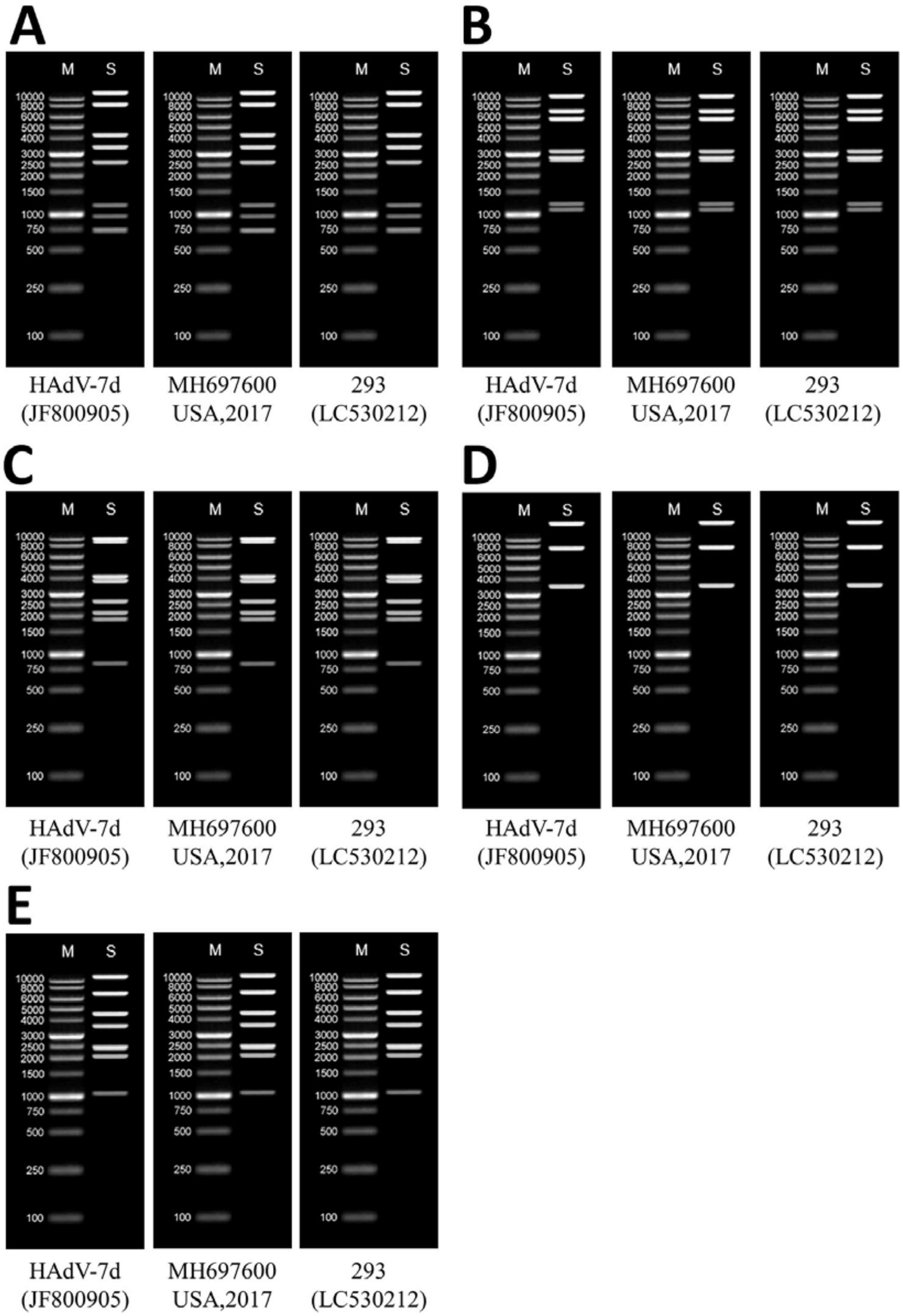

Appendix Figure 2. In silico restriction enzyme cutting pattern. A–E indicate restriction enzyme names used in this analysis: BamHI (A), BclI (B), BstEII (C), HpaI (D), and SmaI (5). HAdV-7d

(JF800905) and MH697600 are reference strains of HAdV genome type 7d, and 293 (LC530212) is isolated strain in this study. In silico analysis with several enzyme were performed with whole-genome sequences of JF800905, MH697600 and LC530212 using online software (http://www.molbiotools.com/restrictionanalyzer.html).
